# Phylogeographic history of grey wolves in Europe

**DOI:** 10.1186/1471-2148-10-104

**Published:** 2010-04-21

**Authors:** Małgorzata Pilot, Wojciech Branicki, Włodzimierz Jędrzejewski, Jacek Goszczyński, Bogumiła Jędrzejewska, Ihor Dykyy, Maryna Shkvyrya, Elena Tsingarska

**Affiliations:** 1 Museum and Institute of Zoology, Polish Academy of Sciences, ul Wilcza 64, 00-679 Warszawa, Poland; 2 Institute of Forensic Research, ul Westerplatte 9, 31-033 Kraków, Poland; 3 Department of Genetics and Evolution, Institute of Zoology, Jagiellonian University, Ingardena 6, 30-060 Kraków, Poland; 4 Mammal Research Institute, Polish Academy of Sciences, 17-230 Białowieża, Poland; 5 Department of Forest Zoology and Game Management, Warsaw University of Life Sciences - SGGW, ul Nowoursynowska 159, 02-776 Warsaw, Poland; 6 Department of Zoology, Biological Faculty, Ivan Franko National University of Lviv, Hrushevskogo str 4, 79005 Lviv, Ukraine; 7 The Schmalhausen Institute of Zoology, National Academy of Sciences of Ukraine, Bohdan Khmelnitsky str 15, 01601 Kyiv, Ukraine; 8 BALKANI Wildlife Society, Str T Tserkovski 67/V - 3, Sofia, Bulgaria

## Abstract

**Background:**

While it is generally accepted that patterns of intra-specific genetic differentiation are substantially affected by glacial history, population genetic processes occurring during Pleistocene glaciations are still poorly understood. In this study, we address the question of the genetic consequences of Pleistocene glaciations for European grey wolves. Combining our data with data from published studies, we analysed phylogenetic relationships and geographic distribution of mitochondrial DNA haplotypes for 947 contemporary European wolves. We also compared the contemporary wolf sequences with published sequences of 24 ancient European wolves.

**Results:**

We found that haplotypes representing two haplogroups, 1 and 2, overlap geographically, but substantially differ in frequency between populations from south-western and eastern Europe. A comparison between haplotypes from Europe and other continents showed that both haplogroups are spread throughout Eurasia, while only haplogroup 1 occurs in contemporary North American wolves. All ancient wolf samples from western Europe that dated from between 44,000 and 1,200 years B.P. belonged to haplogroup 2, suggesting the long-term predominance of this haplogroup in this region. Moreover, a comparison of current and past frequencies and distributions of the two haplogroups in Europe suggested that haplogroup 2 became outnumbered by haplogroup 1 during the last several thousand years.

**Conclusions:**

Parallel haplogroup replacement, with haplogroup 2 being totally replaced by haplogroup 1, has been reported for North American grey wolves. Taking into account the similarity of diets reported for the late Pleistocene wolves from Europe and North America, the correspondence between these haplogroup frequency changes may suggest that they were associated with ecological changes occurring after the Last Glacial Maximum.

## Background

Historical processes during the Pleistocene glaciations had a profound effect on intra-specific genetic differentiation [1-3]. In many extant species, distinct mitochondrial (mt) DNA lineages have non-overlapping geographic distribution, which may result from their isolation in different glacial refugia during the Last Glacial Maximum (LGM, 21,000 - 17,000 years B.P.) [4]. Genetic divergences between these mtDNA lineages are often dated to early Pleistocene or Pliocene [1-5], which may suggest their long-term geographic separation.

However, recent studies based on mtDNA preserved in remains of Late Pleistocene mammals showed that an association between phylogenetic structure and geography does not necessarily imply long-term genetic isolation [6-9]. In cave hyenas *Crocuta crocuta spelaea*, cave bears *Ursus spelaeus*, and brown bears *U. arctos *living in Europe before the LGM no phylogeographic patterns similar to those observed in extant species have been detected [8]. Based on this finding, it has been suggested that current phylogeographic patterns are transient relics of the last glaciation [8]. On the other hand, the study of Late Pleistocene brown bears from eastern Beringia (contemporary Alaska) revealed that a major phylogeographic change occurred 35,000-21,000 years B.P. (hence before the LGM), and the population genetic structure which formed during that time persisted until present [7]. The study of ancient grey wolves *Canis lupus *from Beringia indicated the disappearance of an entire mtDNA lineage at the end of the Pleistocene, and showed that it reflected the extinction of a distinct wolf ecomorph [10]. The loss of one of two major mtDNA lineages has also been revealed in the woolly mammoth *Mammuthus primigenius *about 44,000 years B.P. [11]. All these studies are consistent in showing the high complexity of Quaternary population histories, contrasting with the idea of lineages being geographically fixed throughout subsequent glacial cycles. However, a general mechanism underlying those species-specific population histories, leading to the extinction of some lineages or entire species and the survival of others, still remains unclear [11,12].

In this study, we addressed this question by reconstructing the phylogeographic history of European grey wolves, based on the analysis of mtDNA variability of extant populations and the comparison with ancient data. The Late Pleistocene history of this species has already been studied in North America [10], allowing a comparison between evolutionary histories of the same species on different continents.

## Methods

### Materials

We collected information about mtDNA control region sequences of 947 contemporary wolves from 23 European countries, based on our published [13] and new data (for 674 individuals from 11 countries, altogether) and studies by other authors [14-17] (for details, see Supplementary Data). Given that the sequences analyzed in different studies included different fragments of the control region, only a 230 bp fragment common to all sequences could be compared. Therefore, we sequenced an additional control region fragment using primers L16462 and H222 [18] for 42 wolves carrying each of the 22 European haplotypes detected in Ref. [13] (w1-w22, see Table S1 in Additional file 1), selecting two individuals representing most distant geographic locations of each haplotype, except for haplotypes w18 and w20, each detected in one individual only. Combining this new fragment with the fragment that we had sequenced earlier (see Ref. [13]), we obtained 661 bp control region sequences. We also retrieved GenBank sequences of the same length for three additional European haplotypes (w24, w26 and w27), Indian and Himalayan wolves, as well as coyotes *C. latrans*, which were used as an outgroup (for details, see Additional file 1). These longer sequences were used to perform additional phylogenetic analyses, but could not be used for population genetic analyses due to the limited number of samples sequenced.

To assess the level of distinctiveness of European populations and their contribution to the overall genetic variability of the species, we analyzed the relationships between European wolves and their conspecifics from other continents. For this purpose, we collected mtDNA control region sequence data on contemporary and historical grey wolves from Asia and North America from published studies [14,15,19-23] and NCBI database (Table S1 in Additional file 1). We compared the 230 bp fragment, common to all considered sequences.

We then compared the mtDNA sequences of the contemporary wolves with published sequences of 24 ancient wolves from Europe [GenBank: DQ852634-DQ852637, DQ852643-DQ852662] [24]. These sequences have been produced using laboratory procedures ensuring high reliability of the resulting data (for details, see Ref. [24]). Age of the ancient samples ranged between 44,000 and 1,200 years B.P., and their distribution throughout that period was close to uniform, except for the absence of the samples from the period between 14,000 and 2,000 years B.P. (Figure S1 in Additional file 1). Although the available ancient sequences were short - only 57 bp [24], they included 19 out of 23 parsimony informative sites present in the 230 bp fragment that we analysed for contemporary worldwide wolves (Figure S2 in Additional file 1). Thus, these short sequences were informative enough to infer the phylogenetic position of the ancient wolves relative to their contemporary conspecifics.

### Analysis of phylogenetic relationships among wolf mtDNA haplotypes

We analyzed phylogenetic relationships among (a) contemporary European grey wolves based on a 230 bp control region sequence, (b) contemporary European grey wolves based on an extended 661 bp sequence, (c) contemporary grey wolves worldwide, based on the same 230 bp sequence, and (d) ancient and extant European grey wolves, based on a 57 bp sequence.

Phylogenetic trees were constructed in PAUP 4.0b10 [25], using minimum evolution, maximum likelihood, and maximum parsimony methods. The last two trees were obtained using the heuristic search algorithm. For distance and likelihood-based trees we used a model of nucleotide substitution estimated in Modeltest 3.6 [26]. Confidence in the estimated relationships was determined by calculating bootstrap values with 1000 replicates. The phylogenies were rooted with coyote sequences from GenBank.

Additionally, we constructed Bayesian trees in MrBayes 3.2 [27] using a model of nucleotide substitution estimated in MrModeltest 2.2 [28]. We used a coalescent prior on branch lengths (which involves forcing molecular clock), and default settings for other parameters. For a comparison, we also used an exponential prior on branch lengths. We performed two independent, simultaneous runs with four MCMC chains each. The period until standard deviation of split frequencies for the two independent tree samples fell below 0.01 (indicating convergence to the stationary distribution) was treated as burn-in. The analyses were run as long as this burn-in period constituted the first 10% of generations (i.e. 5,000,000-30,000,000 generations altogether, depending on the sequence set analysed).

We also constructed haplotype networks using the statistical parsimony method implemented in the software TCS [29] and the median-joining method implemented in the software Network 4.510 [30]. TCS network was nested according to the rules described in Templeton *et al*. [31] and Templeton & Sing [32]. Phylogenetic relationships among mtDNA haplotypes of the ancient and contemporary grey wolves was performed using network-based methods only, because of the very short sequence data.

### Analysis of phylogeographic patterns and past population demography

To test whether the analyzed sample set is large enough to properly reflect mtDNA variability of contemporary European wolves, we estimated the total number of wolf haplotypes that can be expected in Europe, using the method of rarefaction curve [33] that plots the cumulative number of haplotypes found with increasing sample size (for details, see Additional file 1). We performed this analysis for all European wolves as well as for Eastern European wolves only (i.e. excluding the Iberian and Apennine populations). We then used the European sample set to compare the geographic distribution and frequencies of main haplotype groups in contemporary and ancient wolves from different parts of Europe.

We applied several coalescent models (constant population size, exponential growth, expansion growth, and Bayesian skyline plot) implemented in the software BEAST 1.4.6 [34] to reconstruct past population dynamics of European wolves. This analysis was based on the alignment of mtDNA sequences of contemporary and ancient wolves (for details on the analysis, see Additional file 1). Because all the ancient haplotypes were closely related to contemporary ones, and three haplotypes were shared between ancient and contemporary samples (see Results), we assumed that ancient and contemporary wolves represent the same population in different time periods. Only the ancient samples with the exact radiocarbon dating (as reported in Ref. [24]) were used in this analysis, and sample ages were fixed to the mean uncalibrated radiocarbon date. Substitution rate was estimated from the data. The plots of historical demographic changes were constructed using TRACER 1.4[35]. To test which of the coalescent models better fits the data, we compared them using Bayes factor values.

Because the use of short sequences (57 bp) available for ancient wolves could bias the results, we performed the same analysis based on the longer contemporary sequences only, for a comparison. In this case, we used a substitution rate of 5 × 10^-8 ^[14], calculated based on sequence divergence between grey wolf and coyote. Using a substitution rate calibrated by divergences between species may lead to an overestimation of recent divergence times [36]. Thus, we expect that the divergence times based on the substitution rate estimated from the ancient data and the fixed one may differ, but if both sets of the analysed data contain enough information to reconstruct the population history, the plots of historical demographic changes should be similar (except for their timing).

Historical demographic expansion suggested by the BEAST analysis was further tested by applying classical population genetic statistics to the modern DNA sequences. Ancient data were excluded from these analyses because of their short length, and because their inclusion could substantially bias the results [37]. We performed the mismatch distribution analysis [38], where we counted each haplotype occurring in a local population only once (to eliminate the bias connected with the likely presence of close kin; see Ref. [39]), but counted the haplotype more than once when it was found in different local populations. We also performed Fu's test of selective neutrality, where a significant negative *Fs *value (at *P *< 0.02) would indicate a demographic expansion [40]. We calculated nucleotide diversity (π), haplotype diversity (H_D_), and the ratio between the number of variable sites (*s*) and the average number of pairwise nucleotide differences (*d*). Populations that experienced recent demographic expansion have high H_D _with low π, and a high *s*/*d *ratio [41]. We applied the software Arlequin 3.1 [42] for these analyses.

## Results

### Phylogenetic relationships between mtDNA haplotypes of contemporary wolves from Europe and other continents

**Figure 1:**
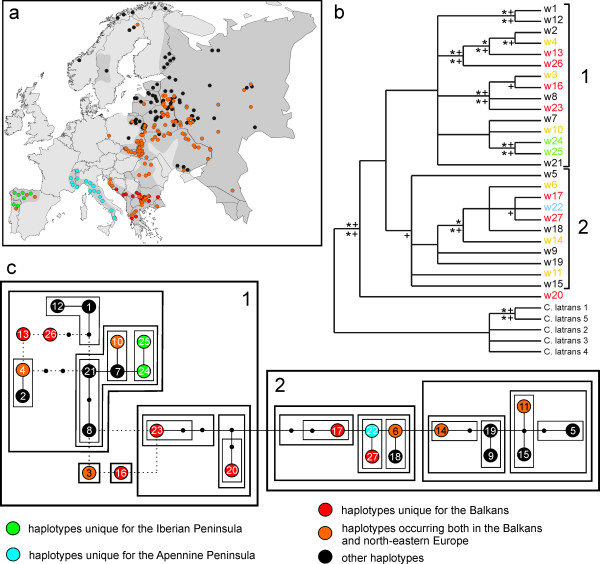
**Spatial distribution and phylogenetic relationships between mtDNA haplotypes of contemporary European wolves**. Based on 230 bp sequences. (a) Distribution of different wolf haplotypes in Europe, against the background of the current wolf range (based on Ref. [54], modified). (b) Maximum parsimony tree (50% majority rule consensus) of mtDNA haplotypes of European wolves. Bootstrap support, if found in more than 50% of 1000 replicates, is indicated as: stars above the branches for the maximum likelihood tree, stars below the branches for the minimum evolution tree, and "+"above the branches for the maximum parsimony tree. "+" below the branches indicate clade credibility values for the Bayesian tree with a coalescent prior, if higher than 0.5. Two main haplogroups correspond to the main clades of the network. Haplotype w20 has an ambiguous haplogroup assignment. (c) Statistical parsimony network of mtDNA haplotypes of European wolves. Large circles represent the haplotypes and small circles indicate interior nodes that were absent from the sample because of insufficient sampling or extinct haplotypes. Each line represents a single mutational change. Dashed lines denote alternative mutational connections. Similar haplotypes are grouped into nested clades, denoted by rectangles.

The comparison of 230 bp mtDNA control region sequences of 947 contemporary European wolves revealed 27 different haplotypes (Table S1 in Additional file 1). Based on the phylogenetic trees and networks constructed, we defined two haplogroups, 1 and 2 (Figure 1 and Figure S3 in Additional file 1). Haplogroup 1 constituted a monophyletic clade, supported by all phylogenetic methods applied (although it had low bootstrap support). All other haplotypes were assigned to haplogroup 2, which was supported as a clade by network-based methods and some tree-based methods, while other methods showed that it consisted of several small clades having a basal position in a tree. In a Bayesian tree constructed with a coalescent prior, haplogroup 2 had a clade credibility support of 0.85. In all the trees and networks constructed, haplotypes from the two groups were clearly separated, although they did not always constitute two monophyletic clades (see Additional file 1 for the discussion on the haplogroup definition). The only inconsistency between different phylogenetic methods concerned the haplogroup assignment of haplotype w20. However, this haplotype was found only in one individual sampled at the edge of the study area (in Turkey), and therefore its possible misassignment would cause only a minor bias in the results. As suggested by most methods, this haplotype was assigned to haplogroup 1. The two haplogroups were separated by five mutational steps: three transitions, one transversion, and one insertion/deletion. The sequence divergence between these haplogroups was 0.030, and the net sequence divergence (accounting for the divergence within haplogroups) was 0.013.

The analysis of longer sequences (661 bp) for 42 selected European individuals revealed 33 haplotypes, two of which have already been reported in GenBank before [accession numbers: AF008139, AF098123, FJ978005-FJ978035]. Additional three European haplotypes of the same length (w24, w26 and w27) were retrieved from GenBank [AF008137, AF098115, and AB007372]. In 11 cases, we found differences in 661 bp sequences between individuals sharing the same 230 bp haplotypes. Two haplotypes deriving from the same short haplotype were marked with A and B extensions, and were closely related, except for haplotypes w7A and w7B (Figure S4 in Additional file 1). The topology of phylogenetic trees confirmed that haplogroup 1 constitutes a monophyletic clade, while haplotypes from haplogroup 2 had a basal position in the phylogeny and formed 2-3 smaller clades (Figure S4 in Additional file 1). Although there was no bootstrap support for the main tree branches, the subdivision into two main haplogroups was consistent with the subdivision based on shorter sequences, and no haplotype assigned earlier to one of the haplogroups was assigned differently when longer sequences were considered. The only exception was haplotype w20, but its assignment was ambiguous earlier as well.

**Figure 2:**
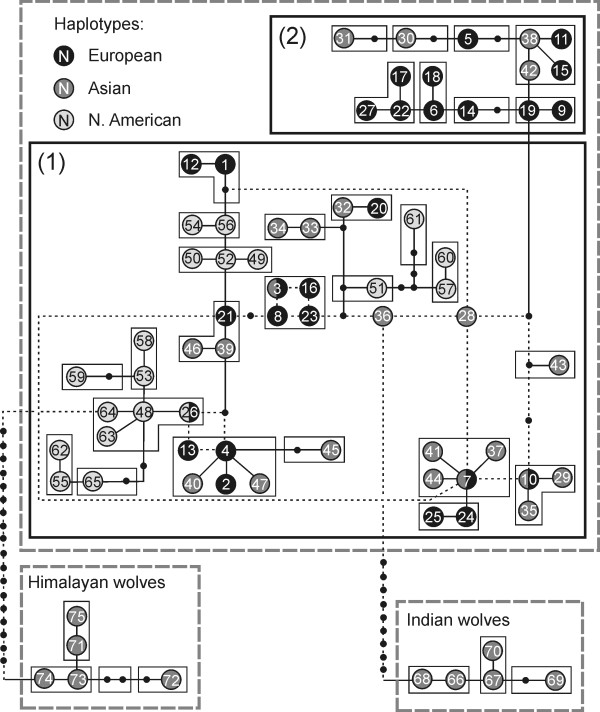
**Statistical parsimony network of mtDNA haplotypes of worldwide grey wolves**. Based on 230 bp sequences. As many alternative mutational connections are present in the network, only the first level clades are presented to make the picture clear. Two dashed line rectangles denote haplotype groups corresponding to clades 1 and 2 from Figure 1c. Haplotypes unique to one continent are marked in one colour, and haplotypes occurring in two continents are marked in two respective colours. Haplotypes of Indian and Himalayan wolves were separated from all the other haplotypes by more than 6 mutational steps, which denoted the threshold of the 95% parsimonious connection.

The comparison of 230 bp control region sequences of grey wolves from the entire range of the species revealed 75 different haplotypes: 23 occurred Europe, 30 in Asia, 18 in North America, 3 in both Europe and Asia, and 1 in both Europe and North America (Table S1 in Additional file 1). Haplotypes of Indian and Himalayan wolves formed clearly distinct lineages (Figure 2), consistent with earlier studies [20,22]. With the exception of Indian and Himalayan wolves, haplotypes from different continents did not group into separate clades, and no evolutionarily significant units (as defined in Ref. [43]) could be distinguished. The distinctiveness of the European haplogroups 1 and 2 was maintained when haplotypes of wolves from other continents were incorporated to the dataset (Figures 2 and S5).

### Phylogeographic history and past population demography of European wolves

Applying the rarefaction curve method [33], we estimated the total number of haplotypes currently occurring in contemporary European wolves at 29 (28.72 ± 1.29; see also Additional file 1). Thus, the analyzed sample, which included 27 out of 29 expected haplotypes, was representative of the European wolf population. Consistently, the same analysis performed for the Eastern European population only (with 24 haplotypes sampled) gave an estimate of 26 haplotypes (25.82 ± 1.38).

**Figure 3:**
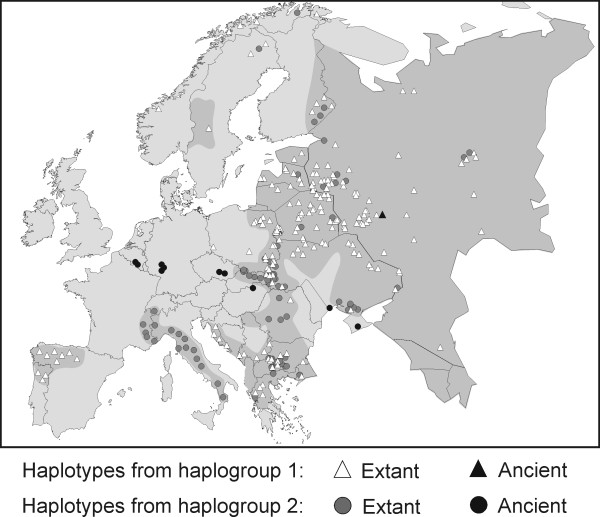
**Distribution of haplogroups 1 and 2 in contemporary and ancient European wolves**. The map is based on the authors' data and the data from other studies [13-17,24].

Geographic distribution of haplotypes of contemporary European wolves did not indicate clear phylogeographic patterns (Figure 1). However, we observed a high frequency of unique haplotypes in southern Europe. In the Apennine Peninsula, only one mtDNA haplotype (w22) occurred, which was unique for this region. In the Iberian Peninsula, two unique haplotypes were found, which occurred in 73% of samples, and in the Balkans, seven unique haplotypes were found that occurred in 45% of samples. Of theremaining 17 European haplotypes, seven were found both in central and north-eastern parts of the continent and in the Balkans (Figure 1a). One haplotype (w10) was common to the Balkans and the Iberian Peninsula, and also occurred in the Caucasus. All haplotypes of contemporary Iberian wolves belonged to haplogroup 1, while the only haplotype of contemporary Apennine wolves belonged to haplogroup 2. In Eastern Europe, these two haplogroups had highly overlapping distributions (Figure 3). Haplogroup 1 predominated in that region, occurring in 87% of individuals.

**Figure 4:**
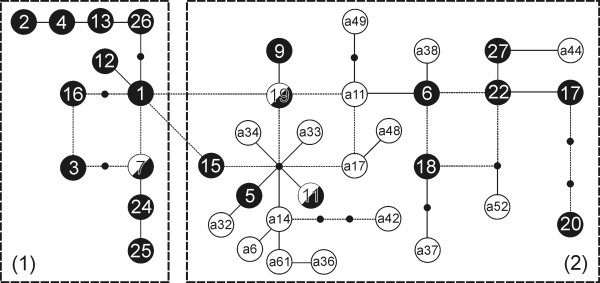
**Statistical parsimony network of mtDNA haplotypes of contemporary (black) and ancient (white) European wolves**. Based on 57 bp sequences. The contemporary haplotypes come from published studies or GenBank (see Table S1 in Additional file 1), and the ancient haplotypes from Stiller *et al*. [24]. Three ancient haplotypes are identical to extant haplotypes (black and white). Thin dashed lines denote alternative mutational connections. Two thick dashed line rectangles denote haplogroups corresponding to clades 1 and 2 from Figure 1c.

In ancient European wolves, haplogroup 2 was predominant. All ancient samples from Belgium, Germany, Czech Republic, Hungary, and Ukraine, ranging in age from 44,000 to 1,400 years B.P., belonged to this haplogroup. Only one haplotype of ancient wolf (w7), sampled in western Russia and dated from 2,700 - 1,200 years ago, belonged to haplogroup 1 (Figures 3, 4 and S6). Assuming contemporary frequencies of haplogroups 1 and 2 in the entire European population (76% and 24%, respectively), the probability that 23 of 24 randomly selected individuals would belong to haplogroup 2 is 1.01 × 10^-13^. This probability would be above 0.05 if the frequency of haplogroup 2 were at least 82.5%. Thus, the predominance of haplogroup 2 in the ancient wolf samples most likely reflects its predominance in the ancient population.

The BEAST analysis based on combined ancient and contemporary data indicated that the most strongly supported model was constant population size over the entire period considered (Bayes factor support against other models was between 5 and 973). The substitution rate estimated based on this model was 2.9 × 10^-6 ^(95% HPDI: 4.9 × 10^-7 ^- 6.4 × 10^-6^). When the BEAST analysis was performed based on the contemporary, longer sequence data only, the most strongly supported model was recent demographic expansion (Bayes factor support against other models was between 1.38 and 10^6^). As a result of the much slower substitution rate applied (5 × 10^-8^), the time scale of density changes was different as compared with the same model for the ancient data (Figures S7a and S7b in Additional file 1). The estimates of the time to the most recent common ancestor (TMRCA) and the effective population size also substantially differed between the two datasets (Table S2 in Additional file 1).

Recent demographic expansion of European wolves suggested by the BEAST analysis of contemporary data was consistent with evidences of expansion inferred from the population genetic parameters. The contemporary European wolf population had high haplotype diversity (0.88), relatively low nucleotide diversity (0.022) and a high *s*/*d *ratio (4.21), consistent with a recent demographic expansion [41]. Consistently, mismatch distribution of all contemporary European haplotypes did not deviate from the expectation of a sudden expansion model (raggedness index = 0.029, *P *= 0.10). Fu's [40] test of selective neutrality indicated a negative, although insignificant *F*_S _value (*F*_S _= -3.88, *P *= 0.18).

## Discussion

We found that with the exception of Indian and Himalayan lineages, contemporary worldwide grey wolves show no evolutionary significant units in terms of reciprocally monophyletic clades with allopatric distributions [43], which is consistent with earlier studies [14,20,22]. Two main haplogroups of worldwide grey wolves, corresponding to the European haplogroups 1 and 2, included both European and Asian haplotypes, and one of them included North American haplotypes as well. Thus, the European haplogroups 1 and 2 did not form any distinct European clade, but they represented a major subdivision within the worldwide wolf population.

We found substantial differences in frequencies of the two haplogroups between contemporary European wolf populations, with haplogroup 1 fixed in the Iberian Peninsula and predominating in Eastern Europe, and haplogroup 2 fixed in the Apennine Peninsula. The Iberian and Apennine populations have very reduced genetic diversity, likely resulting from historical bottlenecks [14,15], and the fixation of a particular haplogroup may result from a strong drift. The fixation of a single haplotype in the Apennine Peninsula prevents conclusions about the intensity of past gene flow between this population and Eastern European population. However, the analysis of contemporary nuclear DNA data suggested that Italian wolves have been genetically isolated for thousands of generations south of Alps [44]. In contrast, the presence of a shared haplotype (w10) between the Iberian Peninsula and Eastern Europe strongly supports past gene flow between these two populations, implying the presence of haplogroup 1 in the extinct intermediate populations from central and western Europe.

Contrary to these expectations, haplotypes of all ancient wolves from central and western Europe, ranging in age from 44,000 to 1,400 years B.P., fall within haplogroup 2. This may reflect historical predominance of haplogroup 2 in central and western Europe for over 40,000 years, both before and after the LGM. Although the ancient sequences were very short, we based our inference on the distribution of haplogroups rather than individual haplotypes, and the analysed data gave us sufficient resolution for this purpose. Our results are consistent with Germonpre *et al*. [45], who showed that the ancient European haplotypes are placed in one part of the wolf haplotype network rather than being scattered across the complete network. Although the haplogroup of the ancient samples from central Europe is consistent with the haplogroup of the most adjacent contemporary wolves from the Carpathian Mountains (see Figure 3), the complete lack of haplogroup 1 in the ancient samples from western Europe is inconsistent with the expectations based on the overall distribution of the haplogroups in contemporary European populations. The lack of haplogroup 1 in the ancient samples is unlikely to result from the sampling bias, as several distant locations in central and western Europe were sampled. Therefore, inconsistency of frequencies of the two haplogroups in the ancient samples with the patterns revealed from contemporary data may suggest substantial changes in haplogroup frequencies over the last 40,000 years from the predominance of haplogroup 2 to the predominance of haplogroup 1.

Parallel haplogroup replacement has been reported for North American grey wolves. Leonard *et al*. [10] showed that mtDNA haplotypes of Pleistocene wolves from eastern Beringia belonged to a distinct haplogroup that does not occur in contemporary North American wolves. This haplogroup corresponds to haplogroup 2 in our study (see Table S3 in Additional file 1), and some of the ancient European and Beringian wolves even shared a common haplotype (haplotype a17 in Figure 4) [10]. The morphological and isotopic data suggest that Beringian wolves were specialized hunters and scavengers of megafauna (preying mainly on horse *Equus lambei *and bison *Bison bison*), and their extinction was connected with the extinction of their megafaunal prey [10]. According to Hofreiter [46], this may imply that Pleistocene wolves across Northern Eurasia and America may have represented a continuous and almost panmictic population that was genetically and probably also ecologically distinct from the wolves living in that area today. The phylogenetic proximity of mtDNA haplotypes alone does not directly imply ecological similarity, but makes it likely, taking into account a significant correlation between genetic and ecological differentiation in contemporary wolves [13,47-49]. Indeed, it has been shown that the late Pleistocene wolves from Belgium that have been genotyped by Stiller *et al*. [24] preyed on horses and large bovids [45], which is consistent with the diet of Beringian wolves [10].

Unlike in North America, haplogroup 2 did not become extinct in Europe. Haplotypes from this group occurred in the wolf samples from western Germany dated for 2,200-1,400 years B.P., and are still present in considerable frequencies in contemporary wolves from Europe and Asia. Although the haplogroup replacement was complete in North America and partial in Europe, the fact that the direction of frequency changes was the same in both cases suggests that they were not random events but were associated with ecological changes occurring after the LGM. If before the LGM haplogroup 2 was exclusively associated with the wolf ecomorph specialized in the megafaunal prey, it would mean that in Europe it was capable to adapt to changing prey composition and availability. Possibly, these changes in Europe were not as substantial and as fast as in North America. For example, the horse - supposedly one of the most important prey species of the Pleistocene wolves [10,45] - did not become extinct in Europe, unlike in North America [e.g. [50]]. Ecological factors, including prey specialization, play an important role in shaping genetic and demographic processes in contemporary populations of wolves and other carnivores [e.g. [13,47-49,51]], and it is likely that they had a similar effect on demographic and evolutionary processes in ancient populations.

Past demographic processes in the European wolf population could be reconstructed only in a limited way. Coalescent Bayesian models (implemented in BEAST) using contemporary data, as well as population genetic statistics like the mismatch distribution suggest demographic expansion of the European wolf population during the last several thousands years (see Figure S7a in Additional file 1). However, the small number of ancient samples and sampling locations, coupled with the lack of contemporary data from north-western Europe due to population extinction, prevent a more detailed insight into the wolf demographic history. The BEAST analysis based on the combined ancient and contemporary data gave the strongest support to the model of constant population size over the entire period considered, which indicates that the available data did not contain sufficient information to infer population history. The timing of the demographic events could not be inferred reliably from the available data, neither, because the short sequences of the ancient samples prevented precise estimates. The substitution rate estimated from the ancient data was very high, which may be due to a very short fragment of the control region studied, which included a large number of variable sites (see Figure S2 in Additional file 1). However, the 95% HPDI of this estimate were broad and overlapped with those estimated for the control region in other mammals and birds based on ancient DNA data [52]. Low number of ancient samples analysed and the presence of population structure may lead to an overestimation of the mutation rate [53], and it may be the case in our study. More detailed reconstruction of the wolf population history in Europe demands the analysis of longer sequence data for a more extensive set of ancient samples with comprehensive spatial and temporal distribution.

## Conclusions

This study, combining the extensive population genetic data on contemporary European wolves with the published data on ancient European wolves, suggests substantial changes in frequency and distribution of two main mtDNA haplogroups throughout the Late Pleistocene and Holocene. These two haplogroups represent a major subdivision within the worldwide wolf population, and co-occur in Eurasia. In North-America, one of these haplogroups was associated with a distinct wolf ecomorph that became extinct at the end of the Pleistocene [10]. We found that the same haplogroup substantially decreased in frequency in Europe since that time. Similarity of population genetic changes in this species that took place in Europe and North America suggests that they may be driven by the same ecological processes associated with the Pleistocene/Holocene transition.

Due to very complex and species-specific population histories of Late Pleistocene mammals, understanding the general mechanism shaping their dynamics is particularly difficult. The comparison of population histories of the same species in different parts of their ranges may be an effective way of approaching this problem, as it provides the possibility of distinguishing general trends from case-specific events. Further comparative studies combining extensive genetic and ecological data on ancient populations are needed to improve our knowledge of the evolutionary processes shaping Late Pleistocene population histories.

## Authors' contributions

MP participated in the study design and laboratory analyses, analysed the data and drafted the manuscript. WB participated in the laboratory analyses, and was involved in revising the manuscript. WJ organised and coordinated the collection of wolf samples from central and eastern Europe. WJ and JG participated in the study design and coordination, supervised the research and were involved in revising the manuscript. BJ, ID, MS and ET were involved in sample collection and participated in revising the manuscript. All authors read and approved the final manuscript.

## Supplementary Material

Additional file 1 **Supplementary data**. Additional file contains details of data collection, and details of some aspects of data analyses. The file also contains a list of all grey wolf haplotypes considered in this study, as well as tables and figures presenting the results which are supportive to the main text. The file is in the PDF format. Click here for file

## References

[B1] Hewitt GM Some genetic consequences of ice ages, and their role in divergence and speciation Biol J Linn Soc 1996 58 247 276

[B2] Hewitt GM The genetic legacy of the Quaternary ice ages Nature 2000 405 907 913 10.1038/35016000 10879524

[B3] Taberlet P Fumagalli L Wust-Saucy AG Cosson JF Comparative phylogeography and postglacial colonization routes in Europe Mol Ecol 1998 7 453 464 10.1046/j.1365-294x.1998.00289.x 9628000

[B4] Avise JC Walker D Johns GG Speciation durations and Pleistocene effects on vertebrate phylogeography Proc Roy Soc Lond B 1998 265 1707 1712 10.1098/rspb.1998.0492 PMC1689361 9787467

[B5] Hewitt GM Post-glacial re-colonization of European biota Biol J Linn Soc 1999 68 87 112 10.1111/j.1095-8312.1999.tb01160.x

[B6] Leonard JA Wayne RK Cooper A Population genetics of Ice Age brown bears Proc Natl Acad Sci USA 2000 97 1651 1654 10.1073/pnas.040453097 10677513 PMC26490

[B7] Barnes I Matheus P Shapiro B Jensen D Cooper A Dynamics of Pleistocene population extinctions in Beringian brown bears Science 2002 295 2267 2270 10.1126/science.1067814 11910112

[B8] Hofreiter M Serre D Rohland N Rebeder G Nagel D Conrad N Münzel S Pääbo S Lack of phylogeography in European mammals before the last glaciation Proc Natl Acad Sci USA 2004 101 12963 12968 10.1073/pnas.0403618101 15317936 PMC516467

[B9] Valdiosera CE García N Anderung C Dalén L Crégut-Bonnoure E Kahlke R-D Stiller M Brandström M Thomas MG Arsuaga JL Götherström A Barnes I Staying out in the cold: glacial refugia and mitochondrial DNA phylogeography in ancient European brown bears Mol Ecol 2007 16 5140 5148 10.1111/j.1365-294X.2007.03590.x 18031475

[B10] Leonard JA Vilà C Fox-Dobbs K Koch PL Wayne RK Van Valkenburgh B Megafaunal extinctions and the disappearance of a specialized wolf ecomorph Curr Biol 2007 17 1146 1150 10.1016/j.cub.2007.05.072 17583509

[B11] Barnes I Shapiro B Lister A Kuznetsova T Sher A Guthrie D Thomas MG Genetic structure and extinction of the woolly mammoth, *Mammuthus primigenius* Curr Biol 2007 17 1072 1075 10.1016/j.cub.2007.05.035 17555965

[B12] Barnosky AD Koch PL Feranec RS Wing SL Shabel AB Assessing the causes of Late Pleistocene extinctions on the continents Science 2004 306 70 75 10.1126/science.1101476 15459379

[B13] Pilot M Jędrzejewski W Branicki W Sidorovich VE Jędrzejewska B Stachura K Funk SM Ecological factors influence population genetic structure of European grey wolves Mol Ecol 2006 15 533 4553 10.1111/j.1365-294X.2006.03110.x 17107481

[B14] Vilà C Amorim IR Leonard JA Posada D Castroviejo J Petrucci-Fonseca F Crandall KA Ellegren H Wayne RK Mitochondrial DNA phylogeography and population history of the grey wolf *Canis lupus* Mol Ecol 1999 8 2089 2103 10.1046/j.1365-294x.1999.00825.x 10632860

[B15] Randi E Lucchini V Christensen MF Mucci N Funk SM Dolf G Loeschcke V Mitochondrial DNA variability in Italian and East European wolves: Detecting the consequences of small population size and hybridization Cons Biol 2000 14 464 473 10.1046/j.1523-1739.2000.98280.x

[B16] Flagstad O Walker C Vilà C Sundqvist A-K Fernholm B Hufthammar AK Wiig O Kojola I Ellegren H Two centuries of the Scandinavian wolf population: patterns of genetic variability and migration during an era of dramatic decline Mol Ecol 2003 12 869 880 10.1046/j.1365-294X.2003.01784.x 12753208

[B17] Valière N Fumagali L Gielly L Miquel C Benoit L Poulle M-L Weber J-M Arlettaz R Taberlet P Long-distance wolf recolonization of France and Switzerland inferred from non-invasive genetic sampling over a period of 10 years Anim Cons 2003 6 83 92 10.1017/S1367943003003111

[B18] Vilà C Savolainen P Maldonaldo JE Amorim IR Rice JE Honeycutt RL Crandall KA Lundeberg J Wayne RK Multiple and ancient origins of the domestic dog Science 1997 276 1687 1689 10.1126/science.276.5319.1687 9180076

[B19] Tsuda K Kikkawa Y Yonekawa H Tanable Y Extensive interbreeding occurred among multiple matriarchal ancestors during the domestication of dogs: evidence from inter- and intraspecies polymorphisms in the D-loop region of mitochondrial DNA between dogs and wolves Genes Genet Syst 1997 72 229 238 10.1266/ggs.72.229 9418263

[B20] Aggarwal RK Kivisild T Ramadevi J Singh L Mitochondrial DNA coding region sequences support the phylogenetic distinction of two Indian wolf species J Zool Syst Evol Res 2007 45 163 172 10.1111/j.1439-0469.2006.00400.x

[B21] Savolainen P Zhang Y Jing L Lundeberg J Leitner T Genetic evidence for an East Asian origin of domestic dog Science 2002 298 1610 1613 10.1126/science.1073906 12446907

[B22] Sharma DK Maldonaldo JE Jhala YV Fleischer RC Ancient wolf lineages in India Proc Roy Soc Lond B 271 2004 3 1 4 10.1098/rsbl.2003.0071 PMC1809981 15101402

[B23] Leonard JA Vilà C Wayne RK Legacy lost: genetic variability and population size of extirpated US grey wolves (*Canis lupus*) Mol Ecol 2005 14 9 17 10.1111/j.1365-294X.2004.02389.x 15643947

[B24] Stiller M Green RE Ronan M Simons JF Du L He W Egholm M Rothberg JM Keates SG Ovodov ND Antipina EE Baryshnikov GF Kuzmin YV Vasilevski AA Wuenschell GE Termini J Hofreiter M Jaenicke-Després V Pääbo S Patterns of nucleotide misincorporations during enzymatic amplification and direct large-scale sequencing of ancient DNA Proc Natl Acad Sci USA 2006 103 13578 13584 10.1073/pnas.0605327103 16938852 PMC1564221

[B25] Swofford DL PAUP Phylogenetic Analysis Using Parsimony and Other Methods 1998 Sunderland, MA, Sinauer Associates

[B26] Posada D Crandall KA MODELTEST: testing the model of DNA substitution Bioinformatics 1998 14 817 818 10.1093/bioinformatics/14.9.817 9918953

[B27] Huelsenbeck JP Ronquist F MRBAYES: Bayesian inference of phylogeny Bioinformatics 2001 17 754 755 10.1093/bioinformatics/17.8.754 11524383

[B28] Nylander JAA MrModeltest v2. Program Distributed by the Author 2004 Evolutionary Biology Centre, Uppsala University, Uppsala

[B29] Clement M Posada D Crandall KA TCS: A computer program to estimate gene genealogies Mol Ecol 2000 9 1657 1660 10.1046/j.1365-294x.2000.01020.x 11050560

[B30] Bandelt H-J Forster P Röhl A Median-joining networks for inferring intraspecific phylogenies Mol Biol Evol 1999 16 37 48 10331250 10.1093/oxfordjournals.molbev.a026036

[B31] Templeton AR Crandall KA Sing CFA A cladistic analysis of phenotypic associations with haplotypes inferred from restriction endonuclease mapping and DNA sequence data. III. Cladogram estimation Genetics 1992 132 619 633 1385266 10.1093/genetics/132.2.619 PMC1205162

[B32] Templeton AR Sing CFA A cladistic analysis of phenotypic associations with haplotypes inferred from restriction endonuclease mapping. IV. Nested analysis with cladogram uncertainty and recombination Genetics 1993 134 659 669 8100789 10.1093/genetics/134.2.659 PMC1205505

[B33] Kohn MH York EC Kamradt DA Haught G Sauvajot RM Wayne RK Estimating population size by genotyping faeces Proc Roy Soc Lond B 1999 266 657 663 10.1098/rspb.1999.0686 PMC1689828 10331287

[B34] Drummond AJ Rambaut A BEAST: Bayesian evolutionary analysis by sampling trees BMC Evol Biol 2007 7 214 10.1186/1471-2148-7-214 17996036 PMC2247476

[B35] Rambaut A Drummond AJ TRACER 1.4 2006 http://tree.bio.ed.ac.uk/software/tracer/

[B36] Ho SYW Shapiro B Phillips M Cooper A Drummond AJ Evidence for time dependency of molecular rate estimates Syst Biol 2007 56 515 522 10.1080/10635150701435401 17562475

[B37] Depaulis F Orlando L Hanni C Using classical population genetics tools with heterochroneous data: time matters! PLoS ONE 2009 4 e5541 10.1371/journal.pone.0005541 19440242 PMC2678253

[B38] Rogers AR Genetic evidence for a Pleistocene population explosion Evolution 1995 49 608 615 10.2307/2410314 28565146

[B39] Hoelzel AR Natoli A Dahlheim ME Olavarria C Baird RW Black NA Low worldwide genetic diversity in the killer whale (*Orcinus orca*): implications for demographic history Proc Roy Soc Lond B 2002 269 1467 1473 10.1098/rspb.2002.2033 PMC1691053 12137576

[B40] Fu YX Statistical tests of neutrality of mutations against population growth, hitchhiking and background selection Genetics 1997 147 915 925 9335623 10.1093/genetics/147.2.915 PMC1208208

[B41] Von Haeseler A Sajantila A Pääbo S The genetical archaeology of the human genome Nature Genet 1996 14 135 140 10.1038/ng1096-135 8841181

[B42] Excoffier L Laval G Schneider S Arlequin ver. 3.0: An integrated software package for population genetics data analysis Evol Bioinf Online 2005 1 47 50 PMC2658868 19325852

[B43] Moritz C Defining "Evolutionarily Significant Units" for conservation Trends Ecol Evol 1994 9 373 375 10.1016/0169-5347(94)90057-4 21236896

[B44] Lucchini V Galov A Randi E Evidence of genetic distinction and long-term population decline in wolves (*Canis lupus*) in the Italian Apennines Mol Ecol 2004 13 523 536 10.1046/j.1365-294X.2004.02077.x 14871358

[B45] Germonpre M Sablin MV Stevens RE Hedges REM Hofreiter M Stiller M Despres VR Fossil dogs and wolves from Palaeolithic sites in Belgium, the Ukraine and Russia: osteometry, ancient DNA and stable isotopes J Archaeol Sci 2009 36 473 490 10.1016/j.jas.2008.09.033

[B46] Hofreiter M Pleistocene extinctions: haunting the survivors Curr Biol 2007 17 R609 R611 10.1016/j.cub.2007.06.031 17686436

[B47] Geffen E Anderson MJ Wayne RK Climate and habitat barriers to dispersal in the highly mobile grey wolf Mol Ecol 2004 13 2481 2490 10.1111/j.1365-294X.2004.02244.x 15245420

[B48] Carmichael LE Krizan J Nagy JA Fuglei E Dumond M Johnson D Veitch A Berteaux D Strobeck C Historical and ecological determinants of genetic structure in arctic canids Mol Ecol 2007 16 3466 3483 10.1111/j.1365-294X.2007.03381.x 17688546

[B49] Musiani M Leonard JA Cluff HD Gates CC Mariani S Paquet PC Vilà C Wayne RK Differentiation of tundra/taiga and boreal coniferous forest wolves: genetics, coat colour and association with migratory caribou Mol Ecol 2007 16 4149 4170 10.1111/j.1365-294X.2007.03458.x 17725575

[B50] Guthrie RD Rapid body size decline in Alaskan Pleistocene horses before extinction Nature 2003 426 169 171 10.1038/nature02098 14614503

[B51] Rueness EK Stenseth C O'Donoghue M Boutin S Ellegren H Jacobsen S Ecological and genetic spatial structuring in the Canadian lynx Nature 2003 425 69 72 10.1038/nature01942 12955141

[B52] Ho SYW Kolokotrinis S-O Allaby RG Elevated substitution rates estimated from ancient DNA sequences Biol Lett 2007 3 702 705 10.1098/rsbl.2007.0377 17785261 PMC2391221

[B53] Miller HC Moore JA Allendorf FW Daugherty GH The evolutionary rate of tuatara revisited Trends Genet 2009 25 13 15 10.1016/j.tig.2008.09.007 18976831

[B54] Boitani L Mech LD, Boitani L Wolf conservation and recovery Wolves: Behavior, Ecology, and Conservation 2003 Chicago, University of Chicago Press 317 340

