# Supplementary material for: Phylogeographic history of grey wolves in Europe
Source: BMC Evol Biol. 2010 Apr 21;10:104. doi: 10.1186/1471-2148-10-104 (PMC2873414; doi:10.1186/1471-2148-10-104)
Supplement: Additional file 1 — Supplementary data. Additional file contains details of data collection, and details of some aspects of data analyses. The file also contains a list of all grey wolf haplotypes considered in this study, as well as tables and figures presenting the results which are supportive to the main text. The file is in the PDF format. [file 1471-2148-10-104-S1.PDF]

ADDITIONAL FILE 1

Phylogeographic history of grey wolves in Europe

Małgorzata Pilot, Wojciech Branicki, Włodzimierz Jędrzejewski, Jacek Goszczyński, Bogumiła Jędrzejewska, Ihor Dykyy, Maryna Shkvyrya, Elena Tsingarska

Details of data collection

Mitochondrial DNA haplotypes of European wolves

The analysis of the frequency and distribution of wolf mtDNA haplotypes in Europe was based on our published (Pilot *et al.* 2006; 643 samples) and new data (31 samples), and other studies on mtDNA variability of European wolves (Vilà *et al.* 1999; Randi *et al.* 2000; Flagstad *et al.* 2003; Valière *et al.* 2003; see Table S1 in Additional file 1). DNA extraction, PCR amplification and sequencing of the new samples were performed as described in Pilot *et al.* (2006). Sequences were compared using BLAST and aligned using Clustal W algorithm (Thompson *et al.* 1994) to identify different haplotypes.

The samples were mapped with various precision, depending on the source of data. Locations of samples from Eastern Europe analyzed in our earlier study (Pilot *et al.* 2006) were determined with the precision of at least 30 km radius. Flagstad *et al.* (2003) reported counties (in Sweden and Norway), and Valière *et al.* (2003) provinces (in France and Switzerland) or countries (Spain, Portugal, Italy, Romania and Croatia) where the samples were collected. Vilà *et al.* (1999) and Randi *et al.* (2000) reported countries only. The samples were mapped with the maximum precision reported in the respective studies. Although sample locations were not precise, this should not affect the analysis made in the scale of entire Europe.

From the studies of Vilà *et al.* (1999) and Randi *et al.* (2000), data on wolves from southern Europe (Spain, Portugal, Italy, Greece, Croatia, former Yugoslavia, Romania) and northern Europe (Finland, Estonia) were used. In the case of haplotypes of wolves from France and Switzerland reported by Valière *et al.* (2003), we used only the haplotypes that were obtained from the analysis of tissue samples, and only from regions where the wolf population is already established, i.e. south-eastern France and southern Switzerland. If there was any premise that the same samples might have been analyzed in more than one study (e.g. the samples of Spanish wolves analyzed by Randi *et al.* (2000) might have been the same as some of the samples analyzed by Vilà *et al.* (1999)), these samples were counted only once.

In the case of wolves from the Scandinavian Peninsula, we used the data from the study of Flagstad *et al.* (2003) on historical, pre-bottleneck wolf population from this region. In particular, we considered the samples from the period 1960-1979. We used these data instead of the data on the contemporary wolf population from Sweden, because Vilà *et al.* (2003) have shown that the contemporary Swedish population had only one female founder. The use of the recent historical data should not cause any bias in the results, because haplotype w1, which is fixed in the contemporary Swedish population, also prevailed in the historical samples from the Scandinavian Peninsula (Flagstad *et al.* 2003).

We also sequenced additional fragment of the control region using the primers L16462 and H222 from Vilà *et al.* (1997) for 42 wolves carrying each of the 22 European haplotypes detected in Pilot *et al.* (2006) (w1-w22, see Table S1 in Additional file 1), selecting two individuals representing most distant geographical locations of each haplotype, except for haplotypes w18 and w20, each detected in one individual only. Combining this new fragment with the fragment that we had sequenced earlier (see Pilot *et al.* 2006), we obtained 661 bp control region sequences. We also obtained from GenBank sequences of the same length for three additional European haplotypes: w24 (AF008137; Vilà *et al.* 1997), w26 (AF098115; F. Koop and co-authors, unpublished) and w27 (AB007372; Tsuda *et al.* 1997), as well as for Indian and Himalayan wolves (AY289973, AY289974, AY289985; Aggarwal *et al.* 2007), and coyotes *C. latrans* (AF008185, DQ480509-DQ480511), which were used as the outgroup.

Mitochondrial DNA haplotypes of Asian and North American wolves

Information about mtDNA control region sequences of contemporary and historical grey wolves from Asia and North America were collected from published studies (Tsuda *et al.* 1997; Vilà *et al.* 1999; Randi *et al.* 2000; Savolainen *et al.* 2002; Sharma *et al.* 2004; Leonard *et al.* 2005; Aggarwal *et al.* 2007) and NCBI database (accession numbers: AF098115-AF098125, AY240073, AY570178-AY570181; see Table S1 in Additional file 1).

Estimation of the expected number of wolf haplotypes in Europe

We estimated the total number of wolf haplotypes that can be expected in Europe, using the method of rarefaction curve (Kohn *et al.* 1999) that plots the cumulative number of haplotypes found with increasing sample size, and estimates the total number of haplotypes as the asymptote of this curve. This method has been originally developed to estimate population size based on the frequency of re-samplings of the same individuals when non-invasive

methods of genetic analysis are applied (Kohn *et al.* 1999), but may be also used to estimate population size (e.g. Leonard *et al.* 2005). Three different functions have been proposed to fit the rarefaction curve. Kohn *et al.* (1999) used the equation: $y = ax/(b+x)$, where y is the cumulative number of haplotypes, x is the number of genotyped samples, a is an asymptote (i.e. estimate of the total number of haplotypes) and b is a slope of the function. Eggert *et al.* (2003) used an equation $y = a(1\text{-}e^{bx})$, and Valière (2002) used an equation proposed by Chessel: $y = a – a[1\text{-}(1/a)]x$ (the meaning of the symbols is the same as in the first equation). As the sampling order affects the curve shape, the data set was randomized 1000 times (without replacement of haplotypes) using GIMLET (Valière 2002), and 1000 rarefaction curves were generated using the R package (Ihaka & Gentleman 1996) and a script file produced by GIMLET. The population size was estimated as the mean value of all iterations for the asymptote a. This procedure was performed for each equation described above.

In the analysis performed for all European wolves, Kohn's equation gave the mean estimate of the total number of haplotypes 28.72 ± 1.29, and median estimate 28.60. Chessel's equation gave the mean estimate 23.15 ± 0.84, and median 23.18. This estimate was lower than the number of sampled haplotypes (27) and therefore unreliable. According to GIMLET manual, Chessel's equation may under-estimate the results, whereas Kohn's equation gives unbiased estimation for medium sampling effort and over-estimation when the sampling effort is high. Eggert's equation could not be fit to the data and therefore did not produce results. Thus, Kohn's equation seems to be most appropriate for the purpose of this study.

Analysis of past population dynamics in BEAST

We applied several coalescent models (constant population size, exponential growth, expansion growth, and Bayesian skyline plot) implemented in the software BEAST 1.4.6 (Drummond & Rambaut 2007) to reconstruct past population dynamics of European wolves. For each model applied, two independent MCMC runs of four chains each were run for 10,000,000 iterations, of which the first 10% was discarded as burn-in. Samples from two runs (which yielded similar results) were combined to estimate model parameters. Genealogies and model parameters were sampled every 1,000 iterations. The strict molecular clock model was applied, the substitution model used was HKY and variation in mutation rates among sites was modelled using a gamma distribution with four rate categories. Mixing and convergence of the chains to the stationary distribution were checked using TRACER 1.4 (A. Rambaut & A.J. Drummond, http://evolve.zoo.ox.ac.uk/software.html?id=tracer). For each run, the effective sample size for each parameter exceeded 100, which indicated efficient

mixing (i.e. low autocorrelation in the Markov chain) and sufficient sampling of model parameters.

Discussion on defining the haplogroups

The two haplogroups defined in this study have been supported in a phylogenetic tree of wolf mtDNA haplotypes constructed by Leonard *et al.* (2007) (see Table S3 in Additional file 1), and there haplogroup 1 formed a clade with a bootstrap support higher than 50%. The study by Vilà *et al.* (1997) showed that the reduction of length of wolf and dog mtDNA control region sequences from 1030 bp to 261 bp decreased the bootstrap support for branches, but did not cause changes in the assignment of haplotypes to the main clades in the wolf/dog tree. This is consistent with a simulation study showing that the analysis of shorter sequence data (500 versus 1000 bp) did not increase the fraction of incorrectly inferred topologies (Woolley *et al.* 2008). Therefore, the consistency of all phylogenetic methods in identifying haplogroup 1 as a clade strongly suggests that this reflects the true topology, despite the lack of bootstrap support for this clade in our study.

References

Aggarwal RK, Kivisild T, Ramadevi J, Singh L: Mitochondrial DNA coding region sequences support the phylogenetic distinction of two Indian wolf species. *J. Zool. Syst. Evol. Res.* 2007, **45**:163–172.

Drummond AJ, Rambaut A: BEAST: Bayesian evolutionary analysis by sampling trees. *BMC Evol. Biol.* 2007, **7**:214.

Ellegren H, Savolainen P, Rosen B: The genetical history of an isolated population of the endangered grey wolf *Canis lupus*: A study of nuclear and mitochondrial polymorphisms. *Phil. Trans. Roy. Soc. Lond. B* 1996, **351**:1661–1669.

Flagstad O, Walker C, Vilà C, *et al*: Two centuries of the Scandinavian wolf population: patterns of genetic variability and migration during an era of dramatic decline. *Mol. Ecol.* 2003, **12**:869–880.

Leonard JA, Vilà C, Wayne RK: Legacy lost: genetic variability and population size of extirpated US grey wolves (*Canis lupus*). *Mol. Ecol.* 2005, **14**:9–17.

Leonard JA, Vilà C, Fox-Dobbs K, Koch PL, Wayne RK, Van Valkenburgh B: Megafaunal extinctions and the disappearance of a specialized wolf ecomorph. *Curr. Biol.* 2007, **17**:1146-1150.

Pilot M, Jędrzejewski W, Branicki W, *et al.*: Ecological factors influence population genetic structure of European grey wolves. *Mol. Ecol.* 2006, **15**:4533–4553.

Randi E, Lucchini V, Christensen MF, *et al.*: Mitochondrial DNA variability in Italian and East European wolves: Detecting the consequences of small population size and hybridization. *Cons. Biol.* 2000, **14**:464–473.

Savolainen P, Zhang Y, Jing L, Lundeberg J, Leitner T: Genetic evidence for an East Asian origin of domestic dog. *Science* 2002, **298**:1610–1613.

Sharma DK, Maldonaldo JE, Jhala YV, Fleischer RC: Ancient wolf lineages in India. *Proc. Roy. Soc. Lond. B* 271, *Biol. Lett. Suppl.* 2004, **3**:1–4.

Stiller M, Green RE, Ronan M, *et al.*: Patterns of nucleotide misincorporations during enzymatic amplification and direct large-scale sequencing of ancient DNA. *Proc. Natl. Acad. Sci. USA* 2006, **103**:13578–13584.

Thompson JD, Higgins DG, Gibson TJ: CLUSTAL W: improving the sensitivity of progressive multiple sequence alignment through sequence weighting position-specific gap penalties and weight matrix choice. *Nucl. Acids Res.* 1994, **22**:4673–4680.

Tsuda K, Kikkawa Y, Yonekawa H, Tanable Y: Extensive interbreeding occurred among multiple matriarchal ancestors during the domestication of dogs: evidence from inter- and intraspecies polymorphisms in the D-loop region of mitochondrial DNA between dogs and wolves. *Genes Genet. Syst.* 1997, **72**:229–238.

Valière N, Fumagali L, Gielly L, *et al.*: Long-distance wolf recolonization of France and Switzerland inferred from non-invasive genetic sampling over a period of 10 years. *Anim. Cons.* 2003, **6**:83–92.

Vilà C, Amorim IR, Leonard JA, *et al.*: Mitochondrial DNA phylogeography and population history of the grey wolf *Canis lupus*. *Mol. Ecol.* 1999, **8**:2089–2103.

Vilà C, Savolainen P, Maldonaldo JE, Amorim IR, Rice JE, Honeycutt RL, Crandall KA, Lundeberg J, Wayne RK: Multiple and ancient origins of the domestic dog. *Science* 1997, **276**:1687–1689.

Vilà C, Sundqvist A-K, Flagstad O, Seddon J, Bjornerfeldt S, Kojola I, Casulli A, Sand H, Wabakken P, Ellegren H: Rescue of a severely bottlenecked wolf (*Canis lupus*) population by a single immigrant. *Proc. Roy. Soc. Lond. B* 2003, **270**:91–97.

Woolley SM, Posada D, Crandall KA: Comparison of phylogenetic network methods using computer simulation. *PLoS ONE* 2008, **3**:e1913.

Table S1. Worldwide distribution of mtDNA control region haplotypes of grey wolf *Canis lupus*

Symbol	Valière	Vilà	Randi	Ellegren	Tsuda	Savolainen	Koop	Sharma	others[a]	Continent	Locality
w1	11	12	7, 8	1						E	Poland, Latvia, Estonia, Belarus, Ukraine
											Russia, Sweden, Norway, Finland
w2										E	Poland, Lithuania, Latvia, Belarus, Ukraine, Russia
w3	9	8								EA	Poland, Latvia, Belarus, Ukraine, Russia, Bulgaria, Saudi Arabia
w4		7	4	4						E	Belarus, Ukraine, Russia, Sweden, Romania, Bulgaria, Greece
w5		13	13	3						E	Latvia, Russia, Finland, Sweden
w6	2		16							E	Poland, Belarus, Ukraine, Russia, Bulgaria, Greece
w7		17			chanco3	15, 18				EA	Belarus, Ukraine, Russia, Saudi Arabia, China
w8										E	Russia, Sweden, Norway, Finland
w9							9, 10			E	Latvia, Russia, Sweden
w10	4	3, 4	9, 10	2						EA	Poland, Russia, Croatia, Bulgaria, Greece, Turkey, Spain, Portugal
w11			17							E	Belarus, Ukraine, Slovakia, Bulgaria
w12										E	Belarus, Russia
w13		10	5		lupus1		2			E	Bulgaria, Greece, former Yugoslavia
w14	8	6								E	Poland, Slovakia, Romania, Bulgaria, Greece
w15										E	Ukraine
w16			1,2							E	Bulgaria
w17										E	Bulgaria, Greece
w18										E	Ukraine
w19			18							E	Poland, Bulgaria
w20										E	Turkey (Trakia)
w21										E	Russia
w22	1	5	14							E	Italy, Switzerland, France

Symbol	Valière	Vilà	Randi	Ellegren	Tsuda	Savolainen	Koop	Sharma	others[a]	Continent	Locality
w23	5		3							E	Croatia
w24	6	1	20							E	Spain, Portugal
w25	7, 10		19							E	Spain
w26	11						1			E, NA	Greece, Canada - Inuvik
w27			15		lupus 2					E	Bulgaria
w28			12							A	Israel
w29		14	11							A	Israel
w30		15								A	Saudi Arabia, India
w31		16								A	Saudi Arabia, India
w32		18							W3Tr, W2Qv	A	Saudi Arabia, Iran, India
w33		19								A	Iran
w34		20								A	Iran
w35		21								A	Afghanistan
w36		22								A	China
w37		23								A	China
w38					chanco2	3				A	China, Mongolia
w39						9				A	China
w40						10				A	China
w41						13				A	China
w42					chanco1					A	Mongolia
w43					chanco4					A	Mongolia
w44					chanco5					A	Mongolia
w45					pallipes					A	Afghanistan
w46									W4Hd	A	Iran
w47									W1Ks	A	Iran

Symbol	Valière	Vilà	Randi	Ellegren	Tsuda	Savolainen	Koop	Sharma	others[a]	Continent	Locality
w48		32					3, 11			NA	Alaska, N-W Territories, Alberta, Montana, Labrador, Inuvik
w49		30								NA	Alaska, Yukon
w50		31								NA	Alaska, Inuvik, Yukon, Montana
w51		33								NA	New Mexico, captive breeding
w52		61					5			NA	Inuvik
w53		38					6, 7			NA	Alaska, Inuvik
w54		37					8			NA	Alaska
w55		28					4			NA	Alaska, Alberta, Inuvik, Minnesota
w56		29								NA	Yukon, Alaska, Inuvik
w57		47[b]								NA	New Mexico
w58		48[b]								NA	Colorado
w59		49[b]								NA	Kansas
w60		50[b]								NA	Nebraska, Oklahoma, Utah, New Mexico
w61		51[b]								NA	New Mexico
w62		52[b]								NA	Oklahoma
w63		53[b]								NA	Utah
w64		54								NA	Labrador
w65									H919	NA	not specified
w66								IWA		A	India
w67								IWB		A	India
w68								IWC		A	India
w69								IWD		A	India
w70									GW-AP1-3[c]	A	India
w71								HWA[b]		A	Nepal
w72								HWB[b]		A	Tibet
w73								HWC	HWTW1-10	A	India - Himalaya
w74								HWD[b]		A	India - Himalaya
w75								HWE[b]		A	Tibet

Based on 230 bp sequences. Numbers and symbols denote haplotype numeration in different studies:
w1 – w75: symbols of haplotypes introduced in this study;
Valière: Valière *et al*. 2003;
Vilà: Vilà *et al*. 1999 and Leonard *et al*. 2005;
Randi: Randi *et al*. 2000;
Ellegren: Ellegren *et al*. 1996 and Flagstad *et al*. 2003;
Tsuda: Tsuda *et al*. 1997;
Savolainen: Savolainen *et al*. 2002;
Koop: F. Koop and co-authors, unpublished (GenBank: AF098115-AF098125);
Sharma: Sharma *et al*. 2004;
[a] other studies: Aggarwal *et al*. 2007, A. Ardalan and co-authors, unpublished (GenBank: AY570178-AY570181), R.L. Gundry and co-authors, unpublished (GenBank: AY240073);
[b] haplotypes found only in museum specimens;
[c] sequences GW-AMM and GW-Gujarat from Aggarwal *et al*. (2007) also match this haplotype;
E – Europe, EA – Eurasia, A – Asia, NA – North America.

Table S2. Results of the BEAST analysis of contemporary and ancient European wolves based on 57 bp sequences of mtDNA control region and estimated substitution rate, and contemporary European wolves based on 661 bp sequences and fixed substitution rate.

Parameters	Contemporary and ancient samples				Contemporary samples			
	Lower 95% HPD limit	Median	Mean	Upper 95% HPD limit	Lower 95% HPD limit	Median	Mean	Upper 95% HPD limit
				Demographic expansion model				
Age estimate (Kyr B.P.)								
All European wolves	45	70	78	136	191	315	325	480
Haplogroup 1	6	56	61	126	144	274	285	441
Haplogroup 2	45	68	74	119	155	291	299	455
10^6 x effective size of modern population ($N_e\tau$)	0.03	1.09	60	304	0.39	4.38	308	126
10^{-6} x substitution rate (subst./site/year)	0.62	2.92	3.43	7.37			0.05	
Mean ln(posterior)			-883				-2013	
Bayes factor*			0.195				1.376	
				Constant population size model				
Age estimate (Kyr B.P.)								
All European wolves	46	83	101	207	204	340	353	521
Haplogroup 1	9	68	83	196	169	301	313	490
Haplogroup 2	46	79	90	165	160	316	325	493
10^6 x effective size of modern population ($N_e\tau$)	0.03	0.09	0.11	0.24	0.42	0.76	0.79	1.21
10^{-6} x substitution rate (subst./site/year)	0.49	2.48	2.91	6.39			0.05	
Mean ln(posterior)			-895				-2017	
Bayes factor*			5.134				0.727	

* Bayes factor of a given model as compared to the second model reported

Table S3. Comparison of the mtDNA haplogroups of worldwide grey wolves from Leonard *et al*. 2007 (based on 421-427 bp) and the present study (based on 230 bp).

Leonard *et al*. 2007	Our data	Geographic location
upper haplogroup	haplogroup 1	
lu1	w24	E
lu2	-	E
lu14	w29	A
lu21	w35	A
lu17	w7	EA
lu23	w37	A
lu4	w10	E
lu8	w3	E
lu9	-	E
lu37	w54	NA
lu18	w32	A
lu19	w33	A
lu20	w34	A
lu22	w36	A
lu33	w51	NA
lu47	w57	NA
lu50	w60	NA
lu51	w61	NA
lu7	w4	E
lu10	w13	E
lu11	-	NA
lu32	w48	NA
lu54	w37	NA
lu53	w63	NA
lu38	w53	NA
lu48	w58	NA
lu49	w59	NA
lu28	w55	NA
lu52	w62	NA
lu29	w56	NA
lu30	w49	NA
lu61	w52	NA
lu31	w50	NA
lower haplogroup	haplogroup 2	
lu5	w22	E
lu6	w14	E
lu15	w30	A
lu16	w31	A

Haplotypes are listed according to the order they appear in the phylogenetic tree in Leonard *et al*. 2007 (Figure 1 therein). Only extant haplotypes from Leonard *et al*. 2007 are listed, together with the corresponding symbols from the current study. The subdivision of the extant haplotypes into the two main haplogroups in the current study strictly corresponds to the subdivision of the extant haplotypes in Leonard *et al*. 2007. The upper haplogroup in the phylogenetic tree in Leonard *et al*. 2007 has a bootstrap support >50%, while the lower haplogroup is unsupported. Sequences of haplotypes lu2, lu9 and lu11 were not submitted to GenBank and therefore they could not be compared with the haplotypes from the present study.

E – Europe, EA – Eurasia, A – Asia, NA – North America.

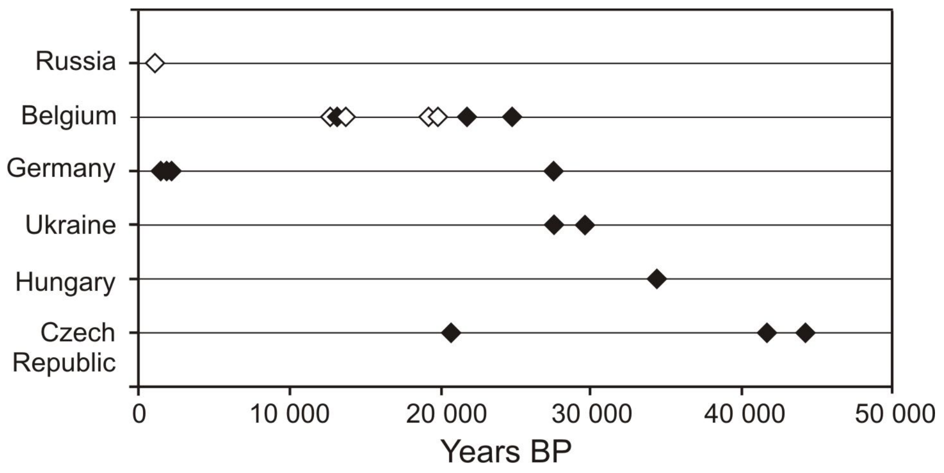

Figure S1. Spatial and temporal distribution of the ancient wolf *Canis lupus* samples. Based on data from Stiller *et al*. (2006). Unfilled symbols denote samples for which only approximate dating was reported.

ACACCCCTACATTCATATATTGAATCACCCCTACTGTGCTATGTCAGTATCTCCAGGTAAAC
CCTTCTTCCCTCCCCTATGTACGTCGTGCATTAATGGTTTGCCCCATGCATATAAGCATGTA
CATAATATTACATTCTTACATAGGACAT**ATTAACTCAATCTCATAATTCACTGATCTATCAA**
CAGTAATCAAATGCATATCACTTAGTCCAATAAGGGCTTAATCA

Figure S2. Sequence of haplotype w1 (230 bp) with parsimony informative sites (when compared with other wolf haplotypes worldwide) indicated in grey. A part of this sequence (57 bp) that was determined for ancient European wolves in the study by Stiller *et al*. (2006) is marked in bold.

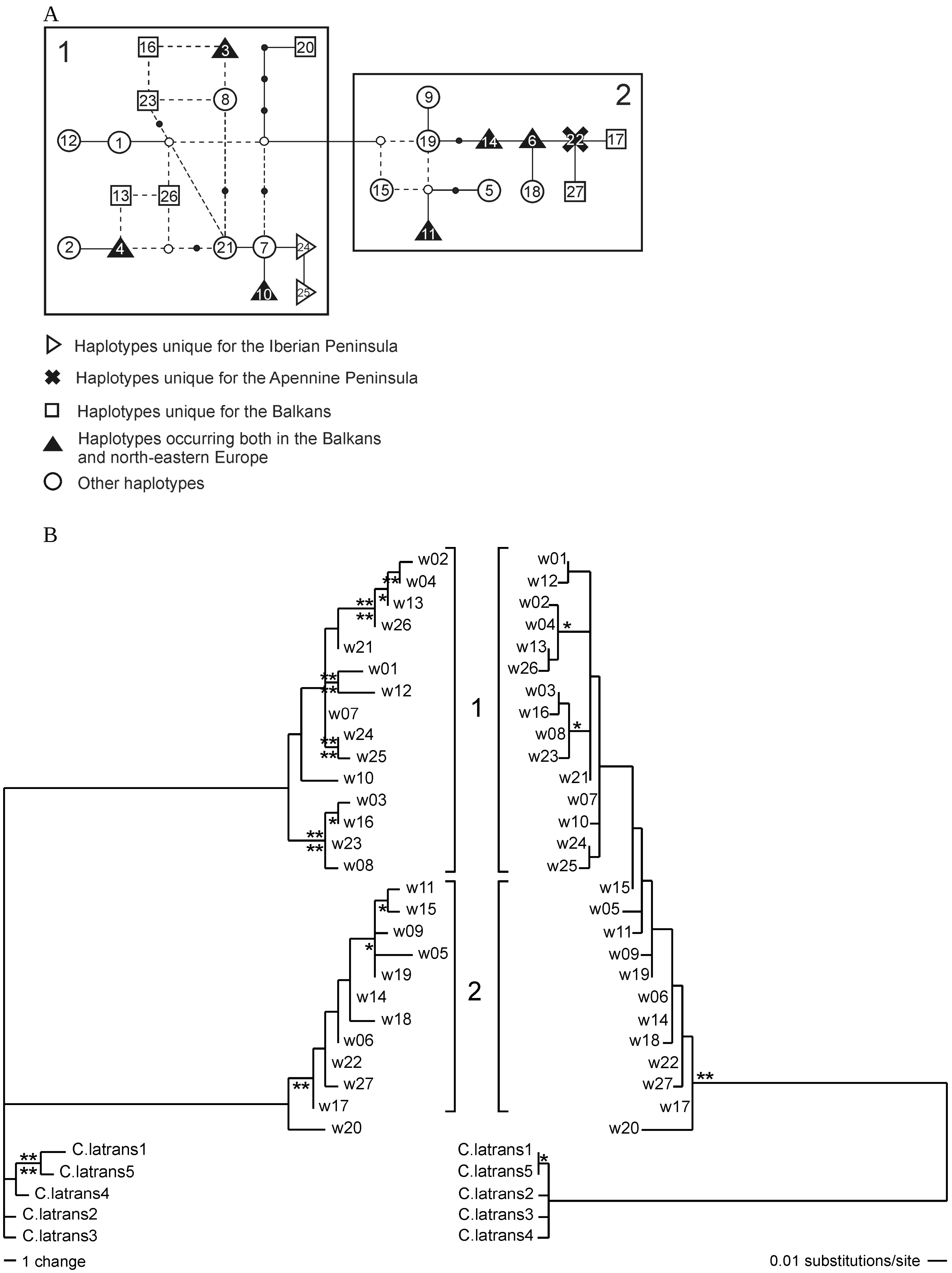
A
1
2
Haplotypes unique for the Iberian Peninsula
Haplotypes unique for the Apennine Peninsula
Haplotypes unique for the Balkans
Haplotypes occurring both in the Balkans and north-eastern Europe
Other haplotypes
B
C.latrans1
C.latrans5
C.latrans4
C.latrans2
C.latrans3
1 change
0.01 substitutions/site

Figure S3. Phylogenetic relationships between mtDNA haplotypes of European wolves (based on 230 bp sequences). A) Median joining network. Large symbols with numbers represent the haplotypes, middle-sized white circles internal nodes, and small black circles mutational transitions. Each line represents a single mutational change. Dashed lines denote alternative mutational connections. B) Left: Bayesian tree constructed using HKY+I+Γ model of nucleotide substitution, and an exponential prior on branch lengths. Clade credibility values are indicated above the branches for an exponential prior and below branches for a coalescent prior (* if >0.5, ** if>0.8). Right: Maximum likelihood tree constructed using TIM+I+Γ model of nucleotide substitution. Bootstrap support is indicated if found in more than 50% of 1000 bootstrap replicates (* if >50%, ** if>80%). The main haplogroups are denoted by numbers 1 and 2.

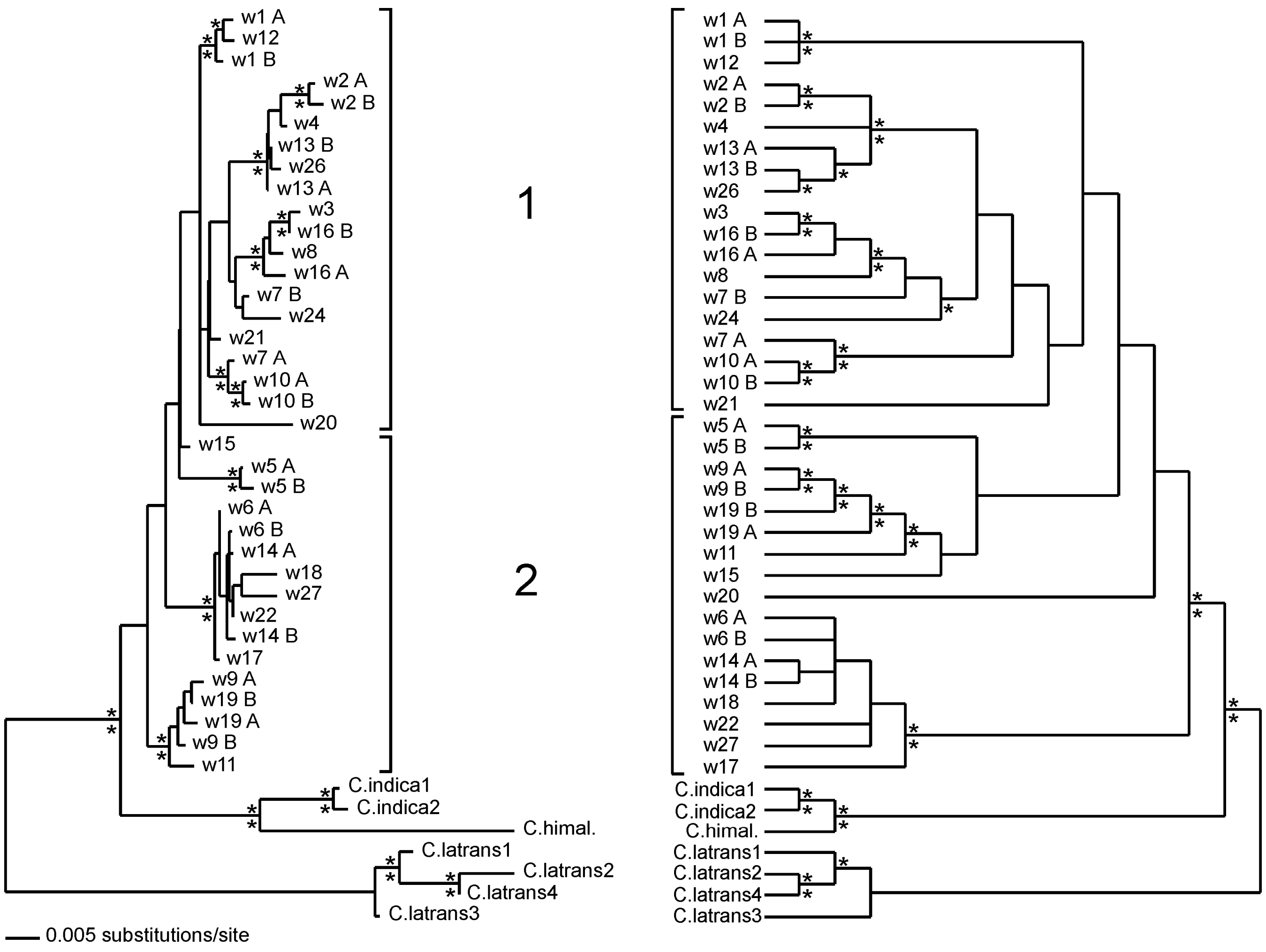

Figure S4. Phylogenetic trees of mtDNA haplotypes of European wolves (based on 661 bp sequences). Left: minimum evolution tree constructed using maximum likelihood distances and HKY+I+Γ model of nucleotide substitution. Bootstrap support for this tree is indicated by stars above branches, and for the maximum likelihood tree below branches, if found in more than 50% of 1000 bootstrap replicates. Right: maximum parsimony tree, 50% majority rule consensus. Bootstrap support for this tree is indicated above branches, and clade credibility values of the Bayesian tree (constructed with a coalescent prior) below branches, if higher than 0.5. Numbers 1 and 2 denote branches corresponding to the main haplogroups from Figure 1 in the main text. Haplotype w20 has an ambiguous position in the phylogeny. The tree is rooted with the sequences of Indian and Himalayan wolves, and coyotes.

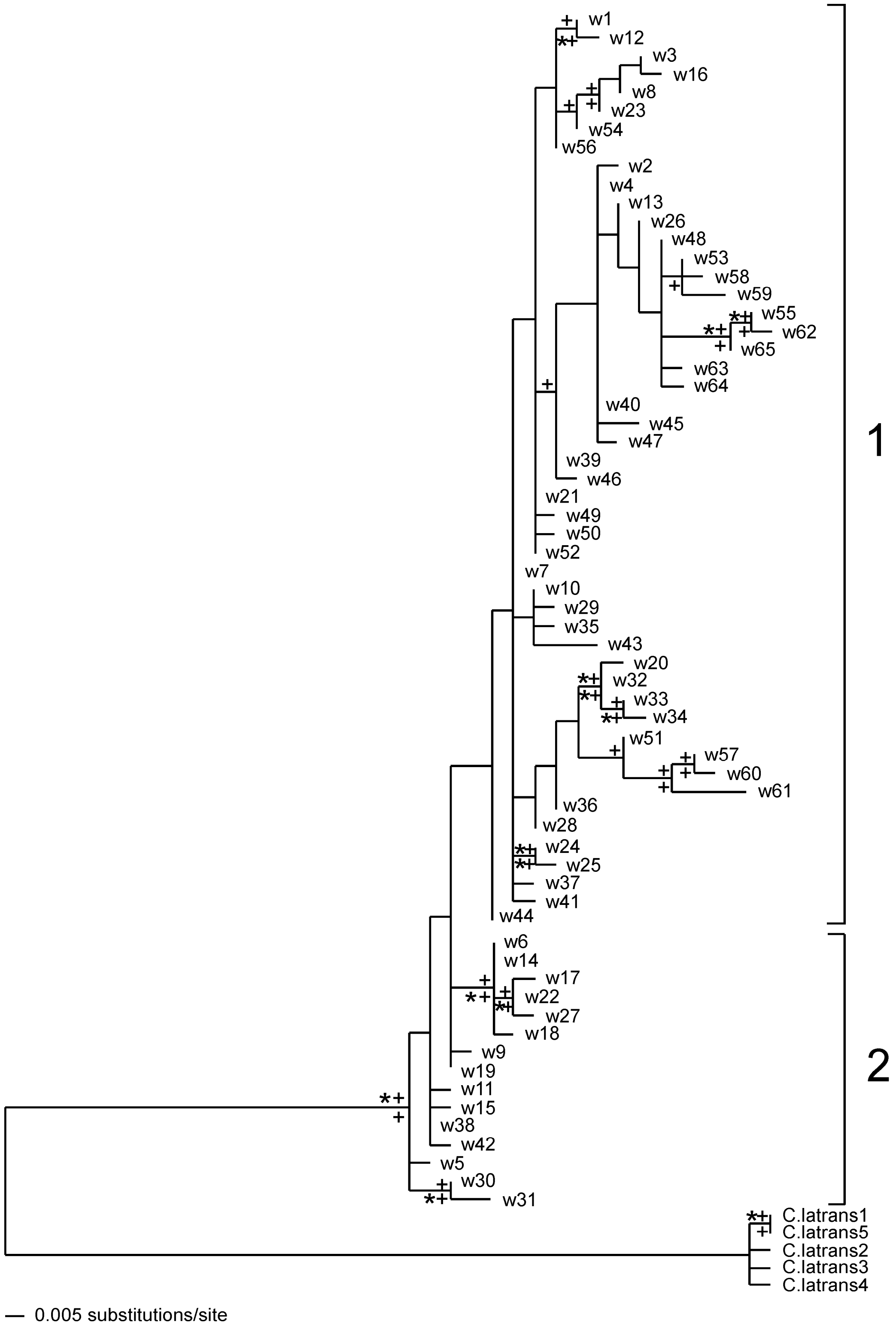

Figure S5. Maximum likelihood tree of worldwide grey wolves based on 230 bp mtDNA sequences, constructed using TIM+I+Γ model of nucleotide substitution. Bootstrap support, if found in more than 50% of 1000 bootstrap replicates, is indicated as: stars above the branches for the maximum likelihood tree, stars below the branches for the minimum evolution tree, and "+"above the branches for the maximum parsimony tree. "+" below the branches indicate clade credibility values for the Bayesian tree (constructed with a coalescent prior) if higher than 0.5. Numbers 1 and 2 denote branches corresponding to the main haplogroups from Figure 1 in the main text.

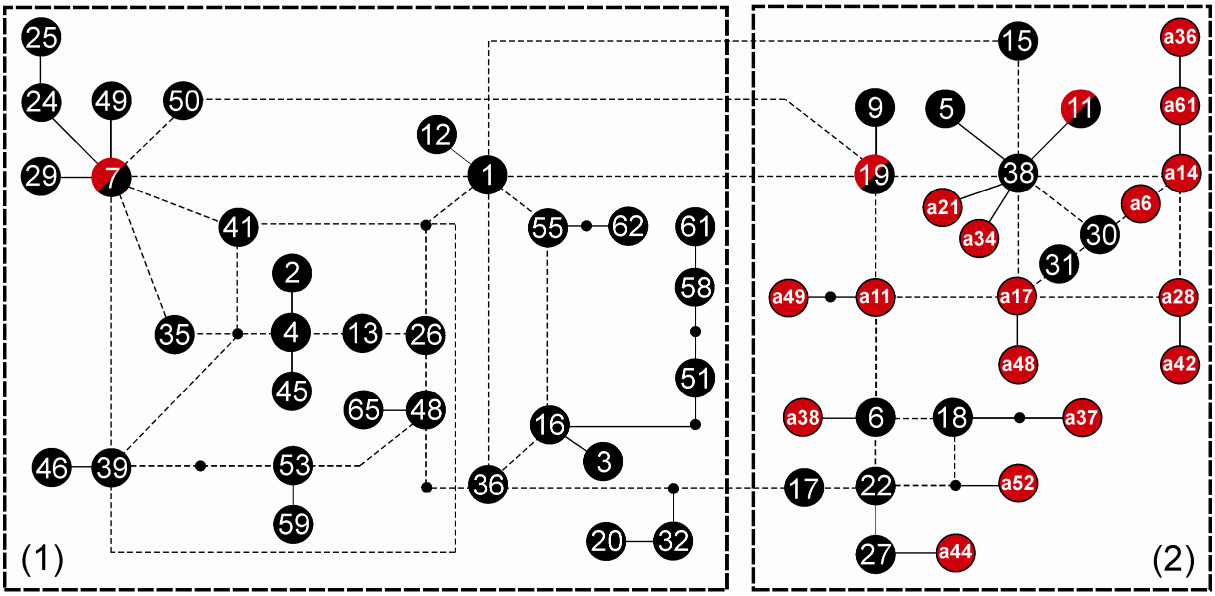

Figure S6. Statistical parsimony network of mtDNA haplotypes of ancient Eurasian wolves (red) and contemporary wolves (black) from the entire species range, excluding India and Himalaya, based on 57 bp common to all sequences.

The haplotypes of contemporary wolves come from published studies and GenBank (see references in Table S1), and the haplotypes of ancient wolves from the study by Stiller *et al.* (2006). Thin dashed lines denote alternative mutational connections. Two thick dashed-line rectangles denote haplogroups corresponding to the European haplogroups 1 and 2.

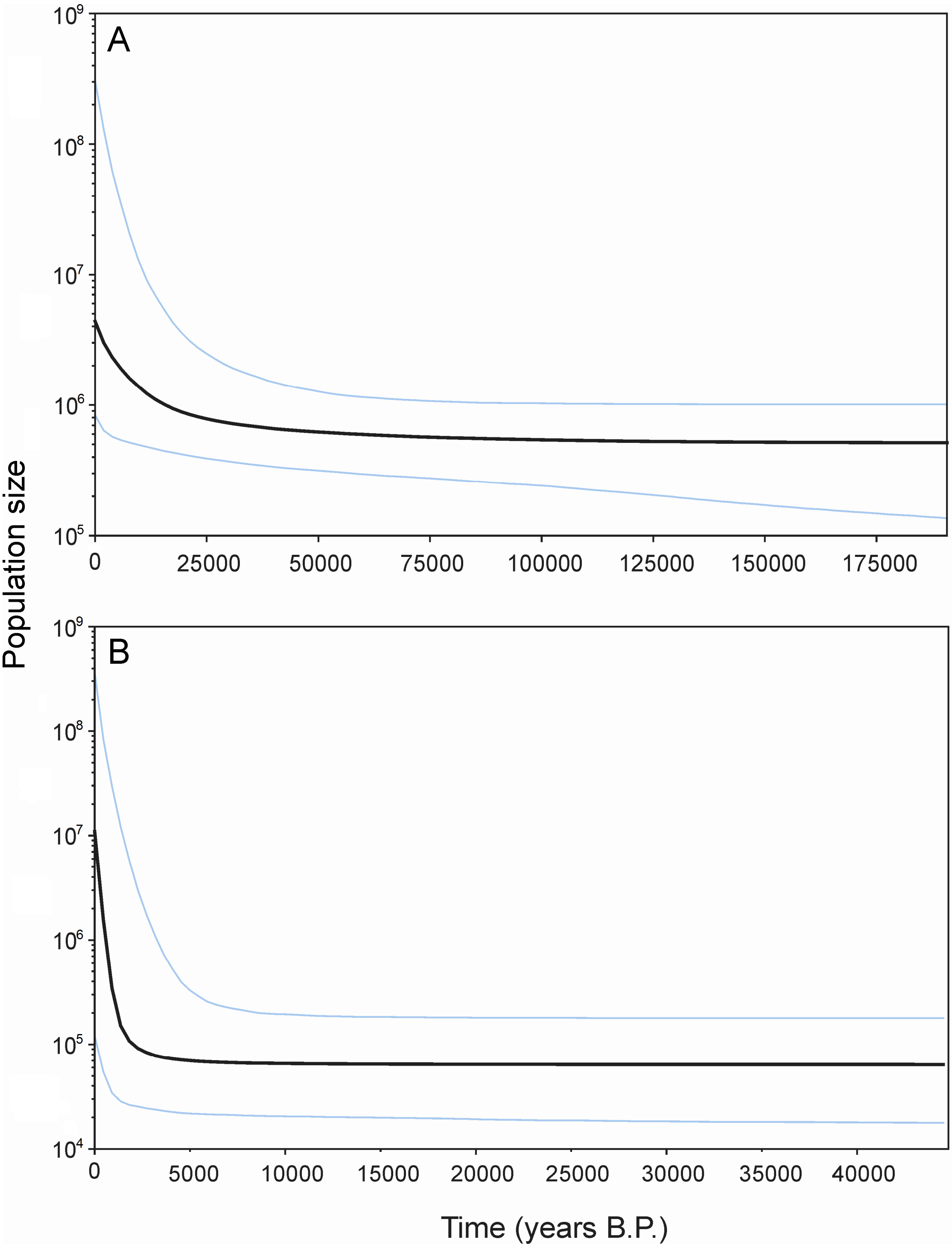

Figure S7. Coalescent reconstruction of past population dynamics of European wolves based on the expansion model implemented in BEAST.

(a) Based on 661 bp sequences of contemporary European wolves, and the fixed substitution rate 5 x 10^{-8}. (b) Based on 57 bp sequences of contemporary and ancient European wolves, and the substitution rate 3.4 x 10^{-6} estimated from the data. The expansion model was the most strongly supported model for the first dataset and the second most strongly supported model for the second dataset (shown here for a comparison). The x-axis units are radiocarbon years, and the y-axis units are a product of the effective population size (N_e) and the generation length in radiocarbon years (τ). The thick black line is the median estimate, and the thin blue lines show the 95% highest posterior density intervals.
